# Threatened preterm labor in Japan: an evidence–practice gap in diagnosis, tocolysis, and guideline implementation

**DOI:** 10.3389/frph.2026.1883392

**Published:** 2026-07-30

**Authors:** Katsuaki Inami

**Affiliations:** Department of Obstetrics and Gynecology, Fujinomiya City General Hospital, Fujinomiya, Japan

**Keywords:** evidence–practice gap, guideline implementation, Japan, preterm labor, ritodrine, threatened preterm labor, tocolysis

## Abstract

Preterm birth remains a major perinatal challenge, and management of women presenting with symptoms suggestive of early labor can shape admission, monitoring, medication use, and transfer pathways. In contemporary international practice, threatened preterm labor is generally managed within a risk-stratified framework, and tocolysis is used mainly for short-term obstetric purposes such as antenatal corticosteroid completion, maternal transfer, and fetal neuroprotection. In Japan, however, threatened preterm labor has often been diagnosed across a relatively broad clinical spectrum and managed with prolonged inpatient observation, bed rest, and extended ritodrine-based tocolysis. This review examines that pattern as a policy and practice problem rather than a narrow therapeutic controversy. It argues that the Japanese approach reflects a persistent evidence–practice gap involving broad diagnostic labeling, limited evidence supporting routine maintenance ritodrine, heterogeneous real-world practice, and guideline wording that remains more permissive than prohibitive. The available literature does not support prolonged maintenance ritodrine as routine care, while associations with maternal and neonatal safety concerns have been increasingly reported. Persistence is interpreted as potentially related to diagnostic breadth, institutional routines, limited therapeutic pathways, and uncertainty about de-escalation, not as evidence of a single cultural or medico-legal cause. This review proposes clearer negative recommendations on prolonged maintenance ritodrine, stronger diagnostic precision, and operational protocols that distinguish high-risk women requiring inpatient care from lower-risk women suitable for reassessment, treatment reduction, or outpatient follow-up.

## 1 Introduction

Preterm birth remains a major global perinatal challenge, with substantial implications for neonatal survival, long-term neurodevelopment, and health systems (1). Despite advances in obstetric and neonatal care, the global burden has not declined sufficiently, and the prevention of avoidable preterm birth continues to be a central objective in maternity care (1). Within this broad context, the management of women presenting with symptoms suggestive of early labor is clinically important because diagnostic labeling and early treatment decisions can shape subsequent admission, monitoring, medication use, and transfer pathways.

### 1.1 Terminology and scope of this review

Terminology in this field is not fully standardized across countries, guidelines, and Japanese observational studies. Terms such as “threatened preterm labor,” “threatened preterm birth,” “preterm labor,” “premature labor,” “acute tocolysis,” “maintenance tocolysis,” and “prolonged ritodrine administration” may refer to overlapping but clinically distinct concepts. To avoid implying that these terms are interchangeable, this review uses the working definitions summarized in Box 1. These definitions are intended to clarify the terminology used in this manuscript and are not proposed as universal diagnostic criteria.

Box 1 **Terminology and exposure definitions used in this review.**

**Table:** 

**Term or phrase**	**Working use and interpretive note in this review**
Symptomatic uterine activity/symptomatic presentation	Symptoms such as uterine contractions, lower abdominal pain, back pain, or pelvic pressure before term, without necessarily indicating cervical change or imminent birth. Symptoms alone are not treated as sufficient evidence of preterm labor or as an automatic indication for prolonged treatment.
Threatened preterm labor/threatened preterm birth	A broad clinical state or diagnostic label in which symptoms suggest possible early labor or risk of preterm delivery, but the probability of imminent birth remains uncertain. “Threatened preterm birth” is retained when referring to Japanese studies that used that term; it is not interpreted as proof of imminent delivery in every case.
Suspected preterm labor	A symptomatic presentation that raises concern for preterm labor before objective confirmation of short-term delivery risk. This term is used mainly when discussing risk-stratified guideline frameworks such as NICE.
Diagnosed or established preterm labor/preterm labor	Higher-probability states in which regular uterine contractions are accompanied by cervical change, or in which objective assessment indicates increased likelihood of preterm birth within a clinically relevant time frame. Diagnosed and established preterm labor are not identical categories in all guidelines, but both are distinguished here from broad symptomatic labeling of threatened preterm labor.
Premature labor/threatened premature delivery	Terms appearing in some Japanese English-language studies, databases, or translated titles. Original source terminology is retained when referring to specific studies, but the manuscript otherwise uses “preterm labor” and “threatened preterm labor” for consistency.
Acute or goal-directed tocolysis	Time-limited tocolytic treatment used to achieve a defined short-term obstetric objective, such as completing antenatal corticosteroids, arranging maternal transfer, or preparing fetal neuroprotection where indicated. This is distinguished from ongoing treatment after the acute objective has been achieved.
Maintenance tocolysis	Continued tocolytic treatment after initial stabilization or after an acute episode, with the intention of maintaining pregnancy beyond the immediate therapeutic window. This is a conceptual treatment category and should not be automatically equated with very prolonged inpatient intravenous ritodrine exposure.
Prolonged ritodrine administration	Extended ritodrine exposure as defined operationally in each study, including use beyond 48 h, treatment for four days or more, or very prolonged inpatient intravenous therapy lasting 28 days or more. These duration thresholds are interpreted separately and are not treated as equivalent measures of the same exposure.
Routine prolonged *β*-agonist-centered management	A practice pattern in which ritodrine or another *β*-agonist becomes part of ongoing default management rather than a short-term, purpose-specific intervention. This is the main target of the review's critique; it does not imply opposition to all acute tocolysis or all inpatient care for women at genuinely high risk.

Two distinctions are especially important for the present review. First, symptomatic uterine activity alone is not treated as equivalent to diagnosed or established preterm labor. In this review, “threatened preterm labor” generally refers to symptomatic presentations or diagnostic labels in which the likelihood of imminent delivery remains uncertain, whereas “preterm labor,” “diagnosed preterm labor,” or “established preterm labor” refers to a higher-risk state supported by cervical change or other objective evidence of short-term delivery risk. Second, “maintenance tocolysis” and “prolonged ritodrine administration” are not treated as identical exposure categories. Maintenance tocolysis refers conceptually to continued treatment after an acute episode or after initial stabilization, whereas prolonged ritodrine administration is an operational exposure definition that varies across studies, including use beyond 48 h, treatment for four days or more, and very prolonged inpatient intravenous therapy lasting 28 days or more. These thresholds are therefore interpreted according to the definitions used in each cited source rather than as equivalent measures of the same exposure.

Against this terminological background, Japan is often regarded as having favorable perinatal outcomes overall. However, the diagnosis and management of threatened preterm labor in Japan show important features that differ from contemporary international practice. As outlined above, many contemporary guidance frameworks distinguish symptomatic presentations from diagnosed or established preterm labor on the basis of cervical change or other indicators of short-term delivery risk. By contrast, studies from Japan suggest that the diagnostic label may be applied across a relatively broad clinical spectrum. In particular, a large nationwide cohort analysis from the Japan Environment and Children's Study, which used the term “threatened preterm birth”, highlighted wide variation in the gestational timing of diagnosis and called for a more unified diagnostic approach (2). The contrast between international and Japanese approaches to threatened preterm labor is summarized in Table 1.

**Table 1:** Selected international guidance frameworks and reported Japanese practice patterns in threatened preterm labor.

Domain	Selected international guidance frameworks/common international framing	Japanese practice patterns reported in the literature	Practical implication
Diagnostic framing	Symptomatic uterine activity is not equated with preterm labor by itself; objective short-term risk assessment is emphasized	Threatened preterm labor/threatened preterm birth may be applied across a broader clinical spectrum	More women may enter an intensive management pathway
Definition of “true” preterm labor	Contractions plus cervical change or clear evidence of imminent delivery	Boundary between symptoms, risk markers, and formal diagnosis may be less sharply defined	Diagnostic labeling may function as an entry point to admission and treatment
Risk stratification	Cervical length, fetal fibronectin, and other tools used to assess likelihood of birth within 48 h	Formal risk stratification may be less consistently embedded in routine framing	Symptoms may carry greater weight than short-term delivery probability
Role of tocolysis	Short-term, purpose-specific treatment	Often embedded in a broader management package	Tocolysis may be used as ongoing management rather than as a time-limited bridge
Main goals of treatment	Antenatal corticosteroids, transfer, fetal neuroprotection	Pregnancy prolongation may become a practical goal beyond the initial acute window	Management may continue even after the acute window
Maintenance tocolysis	Generally not routine; limited support and substantial caution	Reported as historically common, especially with ritodrine	Evidence–practice gap becomes structurally embedded
*β*-agonists	Not preferred or explicitly discouraged in some major guidance frameworks	Ritodrine has remained prominent in practice	Drug choice itself reflects divergence from international norms
Inpatient management	Selective admission based on risk	Admission, bed rest, and prolonged observation have often been reported as combined with tocolysis	Diagnosis, admission, and treatment reinforce one another
Guideline style	Clearer boundaries around what not to offer in routine care	Less explicitly prohibitive wording regarding prolonged maintenance ritodrine	Ambiguity may allow continuation of weakly supported practice

This broad diagnostic framing has been accompanied by a distinctive management pattern. In Japanese obstetric care, threatened preterm labor has long been managed with prolonged inpatient observation, bed rest, and extended tocolytic treatment, especially intravenous ritodrine hydrochloride. National database analyses have shown that prolonged ritodrine administration has been common in routine inpatient care, and that this pattern persisted on a large scale even in the era of evidence-based obstetrics (3, 4). More recent real-world data suggest that the practice may be gradually decreasing, yet it remains substantial and varies according to region and facility type, indicating that long-term ritodrine exposure is not merely a historical phenomenon (4). Key English-language studies describing Japanese threatened preterm labor practice, together with their evidentiary roles and main limitations, are summarized in Table 2.

**Table 2:** Key English-language studies describing Japanese threatened preterm labor practice and evidence limitations.

Study	Design/source	Main focus	Key findings	Evidentiary role and main limitations
Murata et al., 2023 (2)	Nationwide cohort, JECS	Gestational age at diagnosis	Wide variation in timing and likely meaning of diagnosis; supports a more unified diagnostic approach	Broad diagnostic labeling; association-based cohort, diagnostic meaning may vary
Shigemi et al., 2019 (3)	Retrospective cohort, national inpatient database	Inappropriate ritodrine use	Prolonged intravenous ritodrine was common; longer exposure was associated with more maternal adverse events	Practice pattern and safety signal; administrative data, confounding by indication
Ogawa et al., 2025 (4)	Nationwide DPC study	Prolonged ritodrine administration	Prolonged use remained common, though gradually decreasing; marked regional and facility variation persisted	Persistence and institutional variation; operational definition of prolonged use, residual confounding
Murata et al., 2021 (5)	Workshop report	East Asian tocolytic practice	Ritodrine was often used for >48 h in East Asia; unnecessary long-term tocolysis should be avoided	Regional context; workshop report, not population-level evidence
Nakamura et al., 2024 (14)	Maternal mortality analysis	Ritodrine and maternal death	Long-term ritodrine administration was associated with maternal death in reported cases	Serious maternal harm signal; cannot prove causality or estimate absolute risk
Ohashi et al., 2022 (19)	Nationwide claims database	Threatened preterm labor during COVID-19 pandemic	Recorded prevalence of threatened preterm labor and oral ritodrine use decreased during the pandemic	Discretionary diagnostic/treatment zone; indirect pandemic-context evidence
Shibata et al., 2019 (20)	Retrospective hospital-based study	Prolonged hospitalization and fetal growth	Smaller fetal and neonatal anthropometric measures were observed in the prolonged-hospitalization group	Broader management package; retrospective study, cannot separate hospitalization, bed rest, underlying risk, and medication exposure

This persistence is notable because maintenance tocolysis is not supported by prevailing international thinking. A workshop report involving obstetricians from Japan, Korea, and Taiwan noted that ritodrine is often administered for more than 48 h in these settings, although this practice contrasts with several major guidance frameworks that emphasize short-term, purpose-specific tocolysis rather than routine maintenance therapy, and concluded that unnecessary long-term tocolysis should be avoided (5). Likewise, Japanese real-world evidence has raised concern that prolonged ritodrine use may expose women to increased maternal risk without a clearly established corresponding clinical benefit (3). The resulting problem is therefore not simply one of drug choice. Rather, it reflects a broader evidence–practice gap in which diagnostic concepts, therapeutic routines, and institutional habits have remained only partially aligned with international standards.

**Figure 1:**
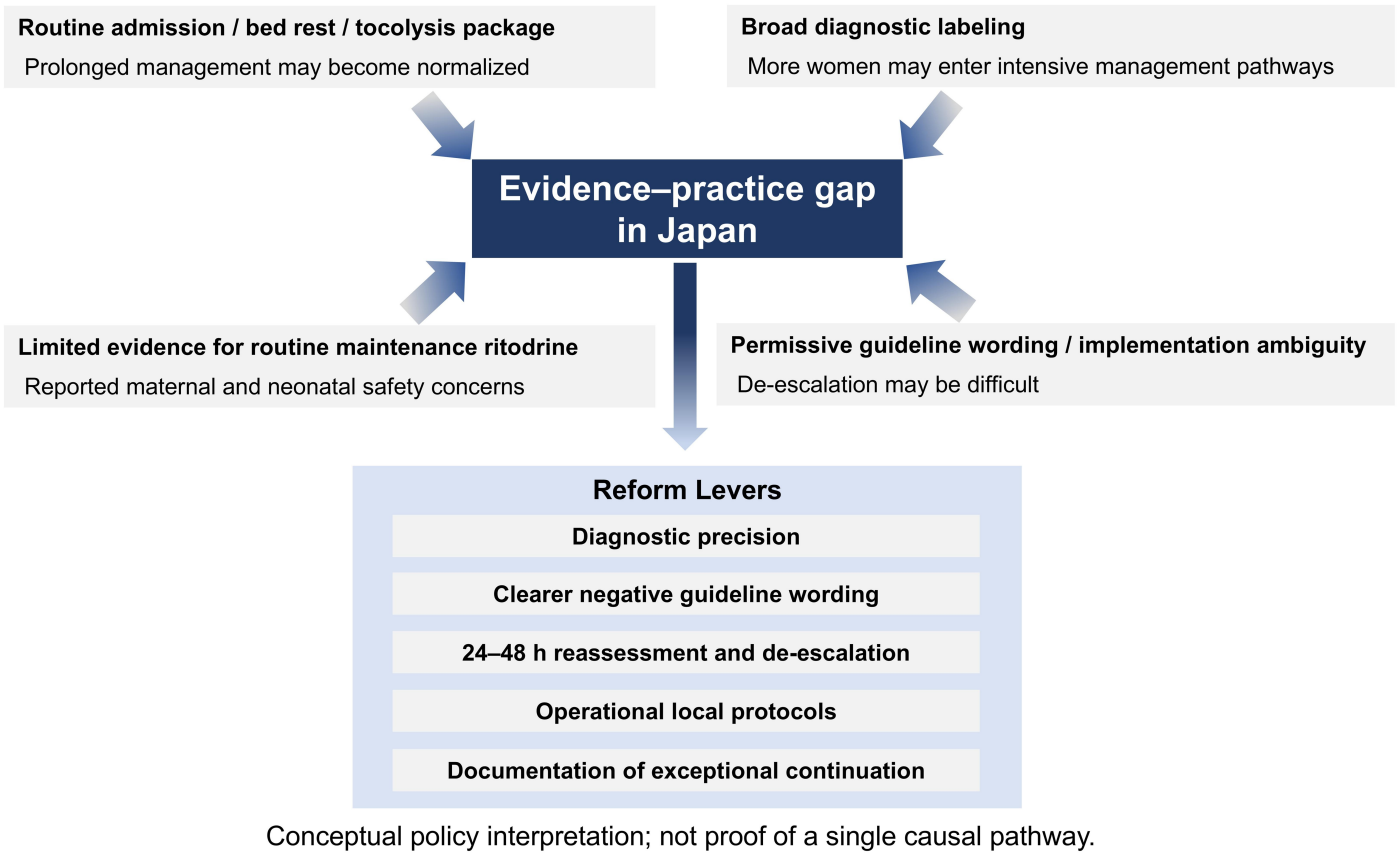
Conceptual framework of possible contributors to the evidence–practice gap in Japan. Broad diagnostic labeling, a routine admission-and-tocolysis management package, limited evidence supporting maintenance ritodrine, and permissive guideline or implementation conditions may interact to sustain an evidence–practice gap in Japan. The framework is intended as a policy interpretation of the literature, not as proof of a single causal pathway. Effective reform requires diagnostic precision, clearer negative guideline wording, and local protocols that make reassessment and de-escalation operational.

The issue also has a policy dimension. When a broad diagnostic label is linked to entrenched management patterns, clinical behavior may be sustained not only by individual preference but also by local protocols, expectations regarding “active” management, and the absence of sufficiently clear negative recommendations. In this setting, ambiguity in guideline interpretation can function as a practical enabler of treatment continuation, even when the evidentiary basis for routine long-term use is weak. A conceptual framework of this evidence–practice gap in Japan is shown in Figure 1.

Accordingly, this review examines threatened preterm labor in Japan not as an isolated therapeutic controversy, but as a policy and practice problem that spans diagnosis, treatment, guideline wording, and implementation. The aim is to clarify how a broad diagnostic concept, routine reliance on prolonged ritodrine-based management, and incomplete translation of international standards into everyday practice have interacted to maintain a persistent gap between evidence and care. By organizing these elements within a single analytical framework, this review seeks to provide the basis for a more coherent discussion of Japanese guideline development and clinical reform.

## 2 Methods

This article was designed as a narrative policy and practice review rather than a systematic review or meta-analysis. No review protocol was registered. The aim was not to produce pooled estimates or an exhaustive inventory of all publications, but to synthesize the evidence and guidance most relevant to the diagnosis, tocolytic management, guideline wording, and implementation of care for threatened preterm labor in Japan.

Relevant literature was identified through targeted searches of PubMed/MEDLINE and Google Scholar, supplemented by backward and forward citation checking of key articles and searches of major guideline and professional society websites. The final search was conducted on 27th June 2026. Search concepts included combinations of the following terms: “threatened preterm labor”, “threatened preterm birth”, “preterm labor”, “premature labor”, “tocolysis”, “maintenance tocolysis”, “ritodrine”, “beta-agonist”, “nifedipine”, “magnesium sulfate”, “bed rest”, “hospitalization”, “Japan”, “Japanese”, “guideline”, “WHO”, “NICE”, “ACOG”, “JSOG”, and “JAOG”. No formal date restriction was applied at the outset; however, current guideline documents and recent nationwide database or claims-based studies were prioritized when assessing contemporary practice. Older randomized trials and meta-analyses were retained when they remained directly relevant to maintenance tocolysis or ritodrine.

Sources were eligible for inclusion if they directly addressed at least one of the following topics: diagnostic framing of threatened preterm labor or preterm labor; acute or maintenance tocolysis; ritodrine or other tocolytic agents; Japanese patterns of diagnosis, hospitalization, or ritodrine use; maternal, fetal, or neonatal safety outcomes related to tocolytic exposure; or guideline and implementation issues relevant to Japanese obstetric practice. For the Japanese practice section, the review focused primarily on English-language peer-reviewed studies to make the evidence base accessible to an international readership. Domestic professional guidance from JSOG/JAOG was also considered because it is central to Japanese guideline implementation. In addition to the English-language JSOG/JAOG guideline publication, current Japanese-language domestic guidance was reviewed to clarify wording on threatened preterm labor and tocolytic treatment. Japanese-language literature and local institutional protocols were not comprehensively searched, and this is acknowledged as a limitation of the review. Studies or documents were excluded if they did not address preterm labor, threatened preterm labor, tocolysis, ritodrine, guideline implementation, or Japanese practice; if they were not accessible in full text or as an official guideline document; or if they provided only indirect background information without relevance to the policy and practice questions considered here.

Guideline documents were selected to represent major contemporary positions relevant to international comparison and Japanese practice. WHO guidance was included because of its global role and its 2022 recommendation on tocolytic therapy. NICE guidance was included because it provides an explicit risk-stratified framework using suspected, diagnosed, and established preterm labor and contains direct negative wording on betamimetic tocolysis. ACOG guidance was included because it represents a major North American professional position on preterm labor diagnosis and management. JSOG/JAOG guidance was included because it defines professional standards for obstetric practice in Japan. These documents were not intended to represent a single homogeneous “international” standard, but to illustrate how major guidance frameworks differ from routine prolonged *β*-agonist-centered management.

Because this was a narrative policy and practice review, no formal risk-of-bias assessment, GRADE evaluation, or quantitative evidence grading was performed. Instead, the strength and limitations of the evidence were considered narratively according to study design and purpose. Randomized trials and meta-analyses were treated as higher-level evidence for the efficacy of maintenance tocolysis; nationwide database and claims-based studies were interpreted as evidence of real-world practice patterns and safety associations, with attention to confounding by indication, disease severity, coding accuracy, institutional variation, and time-related bias; and case reports were used only as signal-generating evidence for rare or biologically plausible adverse outcomes. Policy interpretations regarding culture, medico-legal pressure, and institutional behavior were therefore distinguished from empirical findings and are presented as plausible explanatory mechanisms rather than established causal relationships.

## 3 International standards for threatened preterm labor and tocolysis

### 3.1 How threatened preterm labor is conceptualized outside Japan

In contemporary international guidance, symptomatic uterine activity is not treated as equivalent to preterm labor in itself. Rather, intervention is generally linked to the likelihood of preterm birth within a clinically relevant time frame and to whether short-term treatment is expected to provide meaningful benefit (6–8). This distinction is important for the present review, because the clinical state described here as threatened preterm labor corresponds most closely to symptomatic presentations in which cervical change is absent, uncertain, or not yet sufficient to indicate imminent delivery, whereas preterm labor implies a higher-probability state in which delivery risk is more immediate.

This logic is particularly clear in the NICE guideline on preterm labor and birth. NICE distinguishes between “suspected”, “diagnosed”, and “established” preterm labor, and recommends the use of transvaginal cervical length measurement or fetal fibronectin testing, where appropriate, to estimate the likelihood of birth within 48 h (7). A cervical length greater than 15 mm, or a negative fetal fibronectin result in the relevant setting, supports the view that preterm labor is unlikely; by contrast, a cervical length of 15 mm or less, or a positive fetal fibronectin result, is treated as evidence of diagnosed preterm labor and triggers management accordingly (7). The emphasis is therefore not on symptoms alone, but on stratifying short-term delivery risk so that treatment is directed towards women most likely to benefit.

**Figure 2:**
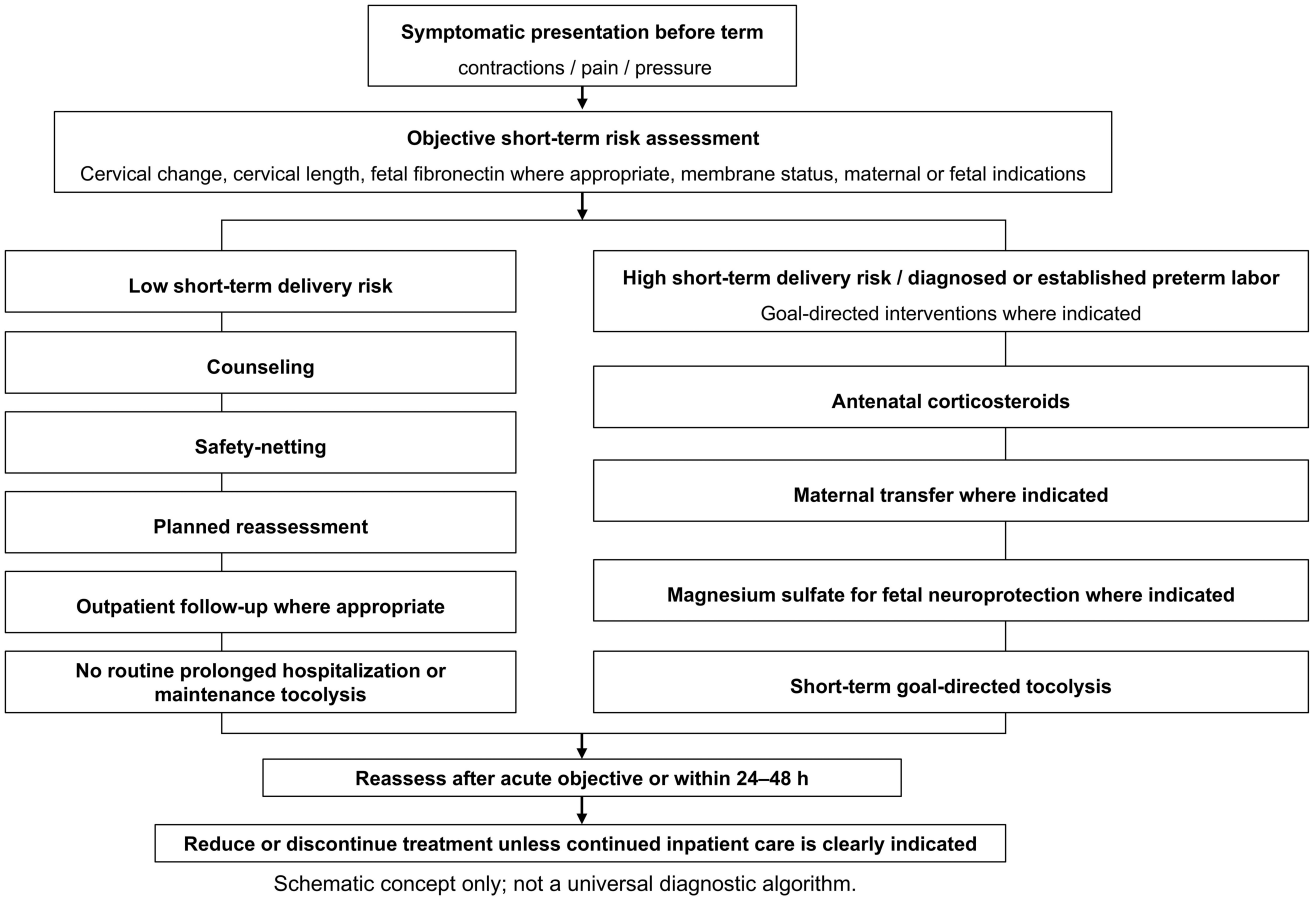
Conceptual distinction between symptomatic threatened preterm labor and diagnosed or established preterm labor in risk-stratified care. The figure is schematic and does not propose universal diagnostic criteria. Symptomatic presentations without clear evidence of imminent delivery should be distinguished from diagnosed or established preterm labor, in which cervical change or other objective findings indicate higher short-term birth risk.

A similar conceptual approach is reflected in North American guidance. ACOG defines the diagnosis of preterm labor largely on clinical grounds, with regular uterine contractions and cervical change occupying a central role, rather than uterine activity alone (8). This does not eliminate diagnostic uncertainty, but it reinforces the principle that preterm labor is more than the mere presence of contractions. In practice, international standards therefore tend to reserve the label of true preterm labor for women in whom symptoms are accompanied by objective evidence that birth may be imminent, while symptomatic women without such evidence are managed within a more explicitly risk-stratified framework. The key conceptual distinction used in this review is summarized in Figure 2.

### 3.2 The international role of tocolysis: short-term bridging rather than open-ended maintenance therapy

Within that framework, the role of tocolysis is generally limited and goal-directed. In many international guidelines, tocolysis is not presented as a means of suppressing contractions indefinitely or of maintaining pregnancy for prolonged periods in routine care. Rather, it is used to create a brief therapeutic window in which other interventions with clearer neonatal relevance can be delivered, most notably antenatal corticosteroids, transfer to an appropriate facility, and, where indicated, preparation for fetal neuroprotection (6–8).

NICE illustrates this approach clearly. It recommends nifedipine for women between 26 + 0 and 33 + 6 weeks of pregnancy who have intact membranes and are in suspected or diagnosed preterm labor, and recommends oxytocin receptor antagonists if nifedipine is contraindicated (7). The same guideline explicitly states that betamimetics should not be offered for tocolysis (7). Magnesium sulfate is addressed separately, not as a preferred tocolytic strategy, but as an intervention for fetal neuroprotection when preterm birth appears likely within 24 h (7). Taken together, this structure makes clear that the objective of intervention is not prolonged suppression of uterine activity as an end in itself, but optimization of the short interval before likely preterm birth.

At the same time, international guidance is not completely uniform, and that nuance should be stated clearly. A 2021 workshop report involving obstetricians from Japan, Korea, and Taiwan noted that maintenance therapy was not supported as routine care in several major guidance frameworks and contrasted this with longer ritodrine use in East Asian practice (5). However, WHO updated its position in 2022. The current WHO recommendation is a context-specific one in favor of nifedipine for *acute and maintenance* tocolytic therapy in women with a high likelihood of preterm birth, provided that specific conditions are met, including gestational age between 24 + 0 and 33 + 6 weeks, absence of contraindications, and the capacity to administer a course of antenatal corticosteroids and/or to transfer the woman to a facility able to care for the preterm infant (6). WHO also states that nifedipine is the preferred option on the basis of the balance of benefits and harms, cost, acceptability, and feasibility (6).

Even so, this updated WHO recommendation should not be misunderstood as support for open-ended or non-selective long-term tocolysis. The recommendation is explicitly restricted to women with a high likelihood of preterm birth, is linked to specific obstetric purposes, and is centered on nifedipine rather than *β*-agonists (6). WHO further notes that commonly used nifedipine regimens in the supporting trials involved an initial oral dose followed by treatment for 3–7 days or until transfer was completed, not indefinite continuation (6). The same document also acknowledges that, although betamimetics can delay birth, they are associated with serious maternal adverse effects that may at times be life-threatening (6). Major international positions on diagnosis and tocolysis are summarized in Table 3.

**Table 3:** Selected major guideline positions on diagnosis and tocolysis in threatened or suspected preterm labor.

Guideline	Diagnostic approach	When to use tocolysis	Drugs/notes	Maintenance therapy	Key message
WHO 2022 (6)	High likelihood of preterm birth required	Nifedipine may be used under specific conditions within a defined gestational window	Nifedipine preferred; serious harms of betamimetics acknowledged	Context-specific support for nifedipine acute and maintenance tocolysis only in women with a high likelihood of preterm birth; no support for open-ended or non-selective long-term treatment	Selective, purpose-linked, high-risk, and nifedipine-centered approach
NICE NG25 (7)	Risk stratification using cervical length/fetal fibronectin	Suspected or diagnosed preterm labor within defined gestational range	Nifedipine preferred; oxytocin receptor antagonist if contraindicated; betamimetics not offered	Does not support routine β-agonist-centered maintenance care	Negative wording helps define non-routine care
ACOG PB171 (8)	Clinical diagnosis centered on contractions plus cervical change	Time-limited use when short-term obstetric benefit expected	Clinical definition emphasized; contractions alone not sufficient	Does not frame tocolysis as an open-ended routine maintenance strategy	Symptoms alone are insufficient; tocolysis is not indefinite

Accordingly, the broad international direction is clear even if details differ between authorities: diagnosis is increasingly tied to risk stratification and objective assessment, and tocolysis is justified mainly when it serves a defined short-term obstetric purpose. Current international standards therefore provide little support for prolonged, routine, *β*-agonist-centered management of threatened preterm labor as a default package of care.

## 4 Evidence on long-term tocolysis and ritodrine: benefit, lack of benefit, and harm

### 4.1 Does maintenance tocolysis prolong pregnancy in a clinically meaningful way?

The evidentiary question in this section is narrower than the general question of whether tocolysis can delay birth at all. As outlined in the previous section, short-term tocolysis is used internationally to create a limited window for antenatal corticosteroids, maternal transfer, and, where indicated, fetal neuroprotection (6–8). Maintenance tocolysis is a different intervention with a different clinical claim: not merely that contractions can be suppressed transiently, but that continued treatment after the acute phase meaningfully improves subsequent maternal or neonatal outcomes. Evidence for that broader claim has remained weak.

This distinction is important because gestational prolongation is not, by itself, a sufficient endpoint. A therapy may delay delivery by several days or even longer without necessarily improving the outcomes that matter most, such as severe neonatal morbidity, mortality, or the need for intensive care. The relevant question is therefore not simply whether pregnancy duration can be extended, but whether extension is clinically meaningful once an initial acute episode has been stabilized. On that point, the literature has generally been unsupportive of routine maintenance therapy (6, 9–11).

A meta-analysis by Sanchez-Ramos and colleagues, which examined randomized trials of maintenance tocolytic therapy after successful short-term tocolysis, found no reduction in recurrent preterm labor, preterm delivery, or adverse perinatal outcome sufficient to support routine maintenance treatment (9). Although this synthesis incorporated different drugs and reflected an earlier therapeutic era, its overall conclusion remains notable: continuation therapy after the acute episode did not demonstrate the substantive perinatal benefit that would justify routine use. More contemporary studies have largely reinforced that conclusion.

The APOSTEL-II randomized trial, for example, examined maintenance nifedipine after 48 h of acute tocolysis and completion of corticosteroids in women with threatened preterm labor. Maintenance treatment did not significantly reduce adverse perinatal outcomes compared with placebo (10). Similarly, an individual participant data meta-analysis concluded that nifedipine maintenance tocolysis was not associated with improved perinatal outcome or pregnancy prolongation and should not be recommended for routine practice (11). These findings do not imply that all prolongation of pregnancy is valueless in every conceivable circumstance. Rather, they indicate that routine continuation of tocolytic treatment after the immediate obstetric window has passed is not supported by robust evidence of neonatal gain.

The same caution applies specifically to ritodrine. A randomized controlled trial of oral ritodrine maintenance after successful intravenous tocolysis found no significant benefit in recurrent threatened preterm labor, delivery before key gestational thresholds, or birth weight, while maternal side effects were more frequent in the ritodrine group (12). This point is particularly relevant for Japan, where ritodrine has often been continued not as a short bridge but as a longer management strategy. Evidence supporting such a routine extension has been limited.

Accordingly, the available literature does not provide a persuasive basis for equating long-term tocolysis with clinically meaningful pregnancy prolongation. Even where some postponement of delivery may occur in selected settings, the more important endpoint—improved neonatal outcome—has not been shown consistently (9–12). This is one reason why international guidance has focused on short-term, purpose-specific use rather than open-ended continuation (6–8).

### 4.2 Maternal adverse effects of prolonged *β*-agonist exposure

If the evidence for benefit is limited, the evidentiary threshold for safety becomes even more important. *β*-agonists such as ritodrine are pharmacologically active well beyond the uterus, and their adverse-effect profile has long been recognized. Maternal tachycardia, palpitations, tremor, arrhythmia, pulmonary edema, hyperglycemia, hypokalemia, rhabdomyolysis, and other complications have all been reported in association with this drug class (3, 13–15). For brief, carefully monitored use, clinicians may judge these risks acceptable in selected circumstances. However, once exposure becomes prolonged, cumulative toxicity and the probability of serious events assume far greater practical importance.

A prospective cohort study by de Heus and colleagues compared adverse drug reactions across tocolytic strategies and found that *β*-adrenoceptor agonists were associated with a substantially higher risk of serious adverse reactions than atosiban (13). The significance of that study lies not only in the relative risk estimate, but in the broader conclusion that *β*-agonist-centered or multiple-drug tocolysis carries a non-trivial safety burden. In other words, the concern is not merely that rare idiosyncratic complications exist, but that the drug class itself is materially less benign than would be acceptable for routine long-term use without compelling evidence of benefit.

Japanese data have made this concern more difficult to dismiss. Shigemi and colleagues, using a national inpatient database, characterized prolonged ritodrine administration in threatened preterm birth as inappropriate and explicitly linked that judgment to the lack of demonstrated long-term efficacy and the established risk of severe maternal adverse effects (3). More recently, Nakamura and colleagues analyzed maternal deaths in Japan and reported an association between long-term ritodrine administration and maternal death, with perinatal cardiomyopathy, cerebral hemorrhage, diabetic ketoacidosis, and pulmonary edema highlighted among the relevant causes (14). Because this was not a randomized study, its findings should be interpreted cautiously and not as proof of simple causation. Nonetheless, the signal is sufficiently serious that it cannot be ignored in a policy discussion about routine care.

A further national database linkage study involving more than 1.8 million pregnant women likewise found that intravenous ritodrine was associated with higher maternal cardiopulmonary risk, supporting the view that the harms of ritodrine are not limited to isolated case reports or exceptional clinical circumstances (15). Taken together, these findings suggest that prolonged *β*-agonist exposure should not be regarded as a neutral default from which only small incremental harms arise. Rather, it is a management choice that carries recognized systemic risk.

This matters particularly because long-term tocolysis is often justified by a precautionary instinct: the belief that continuing infusion is safer than stopping it. Yet when the intervention itself has a documented profile of potentially severe maternal complications, continuation cannot automatically be assumed to be the conservative choice. In that respect, prolonged ritodrine may represent not a low-risk extension of acute therapy, but a qualitatively different exposure whose harms become harder to justify as duration lengthens (3, 13–15).

### 4.3 Fetal and neonatal concerns

The evidentiary problem is not confined to maternal safety. Ritodrine also crosses the placenta, and fetal or neonatal consequences have been reported, especially when exposure is prolonged or continues close to delivery (16–18). These concerns do not establish that every exposed infant will experience harm, but they do weaken any assumption that long-term therapy is a maternal issue alone.

Shimokawa and colleagues reported that maternal intravenous ritodrine administration was associated with a markedly increased incidence of neonatal hypoglycemia, and identified maternal age and short interval from cessation of infusion to delivery as relevant risk factors (16). Yada and colleagues, using a Japanese nationwide retrospective cohort, similarly found that neonatal hypoglycemia was associated with ritodrine exposure and that long-term use and cessation directly before delivery were linked to this outcome; the same study also found an association between concomitant ritodrine and magnesium sulfate exposure and critical neonatal hyperkalemia (17). These findings are especially relevant in Japanese practice, where magnesium sulfate may also be used and prolonged tocolysis can extend the duration of fetal exposure.

In addition, case-based literature has described neonatal rebound hyperkalemia after maternal long-term ritodrine administration even without concomitant magnesium sulfate, reinforcing that clinically important neonatal electrolyte disturbance is biologically plausible (18). Case reports cannot define incidence, but they do show that neonatal complications are not purely hypothetical. Once such harms are credible and at least partly documented, prolonged routine administration becomes harder to justify in the absence of clear neonatal benefit.

### 4.4 Why the evidence does not support maintenance ritodrine as routine care

When the evidence is considered as a whole, a consistent picture emerges. First, the rationale for acute tocolysis cannot simply be extrapolated into a rationale for prolonged maintenance treatment (6–8). Second, routine continuation after initial stabilization has not shown convincing improvement in meaningful neonatal outcomes, whether examined in meta-analysis, randomized trials, or drug-specific studies (9–12). Third, *β*-agonist exposure is associated with important maternal harms, including rare but potentially catastrophic events, and recent Japanese analyses have intensified concern about the safety of long-term ritodrine (3, 13–15). Fourth, fetal and neonatal complications, particularly hypoglycemia and hyperkalemia, make clear that prolonged exposure is not harmless on the infant side either (16–18).

Accordingly, the available literature does not support maintenance ritodrine as routine care for threatened preterm labor. This conclusion does not deny that tocolysis may have a legitimate short-term place for defined obstetric goals, nor does it imply that every individual decision to continue treatment briefly is irrational. It does mean, however, that prolonged ritodrine-based management lacks the evidentiary balance of benefit over harm required for a default standard of care (6, 9–18).

The remaining question is therefore not whether the evidence base is sufficient to justify routine long-term ritodrine use, but why the practice has nevertheless remained embedded in Japanese obstetric care. That evidence–practice gap is examined in the next section.

## 5 Japanese practice: A persistent evidence–practice Gap

### 5.1 Broad diagnosis and frequent labeling of threatened preterm labor in Japan

A central feature of Japanese practice is that the diagnostic label of threatened preterm labor appears to be applied across a relatively broad clinical spectrum. As noted in Section 3, contemporary international standards tend to reserve the diagnosis of preterm labor for women with symptoms plus objective evidence of short-term delivery risk, while symptomatic women without clear cervical change are managed within a more explicitly risk-stratified framework (7, 8). In Japan, however, the boundary between mild symptoms, perceived risk, and formal diagnostic labeling has often been less sharply defined.

This point is supported by the Japan Environment and Children's Study analysis by Murata and colleagues (2). That nationwide cohort study examined the association between gestational age at diagnosis of “threatened preterm birth” and subsequent preterm birth, and highlighted substantial variation in the timing and probable meaning of the diagnosis across pregnancy (2). The authors concluded that a more unified diagnostic approach was needed (2). Their findings are important not merely as an epidemiological observation, but because they suggest that the label may function in practice as a broad entry point into surveillance and treatment rather than as a narrowly defined indicator of imminent delivery risk.

Additional indirect support comes from the nationwide claims-based analysis by Ohashi and colleagues during the COVID-19 pandemic (19). That study found that the recorded prevalence of threatened preterm labor fell during the pandemic, while oral ritodrine use also declined significantly (19). By contrast, intravenous ritodrine use did not change significantly (19). Although these findings do not establish causation, they are consistent with the view that a substantial proportion of diagnosed threatened preterm labor in Japan may represent milder or more discretionary presentations, in which diagnostic labeling and treatment initiation are sensitive to behavioral and contextual factors. In that sense, the breadth of diagnosis is not a minor semantic issue. It determines how many women move from symptoms or risk markers into a more intensive management pathway.

### 5.2 Long-term ritodrine remains common in real-world Japanese obstetric care

Once women enter that pathway, long-term ritodrine administration remains a prominent feature of care. In interpreting this literature, the duration thresholds used to define prolonged or maintenance treatment should be kept distinct. The threshold of more than 48 h generally identifies treatment extending beyond the usual acute tocolytic window or beyond the immediate bridging purpose of tocolysis. The threshold of four days or more, used in a recent nationwide DPC analysis, is an operational database definition of prolonged ritodrine administration and captures a broader range of continued treatment. By contrast, inpatient intravenous exposure lasting 28 days or more represents a much more prolonged category. These thresholds therefore indicate different degrees of continuation and should not be interpreted as equivalent measures of the same exposure. The study by Shigemi and colleagues, based on a national inpatient database of 134,959 women admitted with threatened preterm birth, showed that prolonged intravenous ritodrine treatment was not exceptional but common (3). In that cohort, 17.2% received intravenous ritodrine for 48 h or less, whereas 28.7% received it for 28 days or more, and longer duration was associated with higher maternal adverse-event rates during hospitalization (3). These findings are difficult to reconcile with a model in which *β*-agonist treatment is used only briefly for a defined obstetric purpose.

More recent nationwide data indicate some movement away from the most prolonged patterns, but not a fundamental shift to an exclusively short-term strategy. Ogawa and colleagues analyzed 280,734 women in the Diagnosis Procedure Combination database and defined prolonged ritodrine administration as treatment for four days or more (4). Even with that relatively modest threshold, 180,674 women met the definition of prolonged treatment (4). The authors also found that prolonged administration gradually decreased over time, but remained significantly associated with region and type of facility, being more common in primary hospitals than in tertiary hospitals and more frequent in several regions than in Tokyo (4). This variability is important. It suggests that long-term ritodrine use is shaped not only by patient condition but also by institutional norms, local practice patterns, and regional clinical environments.

The 2021 workshop report involving Japan, Korea, and Taiwan similarly observed that ritodrine is often used for more than 48 h in East Asian practice, despite the lack of support for maintenance therapy in several major guidance frameworks (5). Read together, these sources indicate that long-term ritodrine use in Japan should not be interpreted as sporadic deviation from a short-term standard. Rather, it has functioned as an embedded real-world practice pattern, with some recent attenuation but substantial persistence.

Read together, these sources should not be interpreted as showing a single uniform exposure category. Rather, they show that ritodrine continuation in Japan has occurred across a spectrum, from treatment beyond the acute 48-hour window to operationally defined prolonged use of four days or more and, in a substantial subgroup, very prolonged inpatient intravenous therapy lasting 28 days or more. This persistence matters because it transforms the evidence issue reviewed above into a practice issue. The problem is no longer simply whether maintenance ritodrine lacks convincing benefit. It is that treatment strategies with limited evidentiary support have remained integrated into routine hospital care at scale, although the clinical meaning of “prolonged” varies across studies (3–5).

### 5.3 Bed rest, prolonged hospitalization, and the Japanese management package

The Japanese evidence–practice gap cannot be reduced to ritodrine alone. In routine care, threatened preterm labor has often been managed as a package that includes admission, activity restriction or bed rest, prolonged observation, and stepwise pharmacological treatment rather than as a narrowly targeted short-term intervention (3, 19). This critique should not be understood as opposition to inpatient care itself. Hospitalization, transfer, close monitoring, antenatal corticosteroids, fetal neuroprotection, and short-term tocolysis may be clinically appropriate for women with objective evidence of high short-term delivery risk or other maternal or fetal indications. The concern addressed here is narrower: the routine extension of inpatient, activity-restrictive, and pharmacological management for broadly labeled threatened preterm labor when imminent delivery risk remains uncertain.

The study by Ohashi and colleagues is informative in this respect. During the COVID-19 pandemic, oral ritodrine use decreased significantly, whereas intravenous ritodrine use did not (19). The authors interpreted this pattern as suggesting a reduction in milder threatened preterm labor managed without the need for intensive intravenous treatment (19). Although indirect, this finding is compatible with the existence of a broad management zone in which hospital attendance, diagnostic labeling, oral medication, and behavioral restriction may be closely linked.

More directly, Shibata and colleagues examined women with threatened preterm labor who underwent prolonged hospitalization including bed rest but ultimately delivered after 36 weeks (20). Compared with controls, fetal abdominal circumference and estimated fetal weight at later gestation were smaller, and neonatal anthropometric parameters at birth were also smaller in the hospitalized group (20). This study was retrospective and cannot establish that bed rest or hospitalization caused impaired fetal growth. It also cannot isolate the independent effects of admission, activity restriction, underlying obstetric risk, medication exposure, or other features of care. Nevertheless, it is relevant to this review because it illustrates that prolonged hospitalization and bed rest should not be treated as neutral background conditions whose benefits can be assumed.

Accordingly, the Japanese practice pattern should be understood as a package of care in which broad diagnosis, prolonged admission, activity restriction, and sustained tocolysis may reinforce one another in some settings. The policy issue is not whether high-risk women should be admitted or monitored when clinically indicated. It is whether broadly diagnosed threatened preterm labor should routinely lead to prolonged inpatient management when the evidence supporting that package is limited and when its physiological, psychological, social, and economic burdens may be substantial.

### 5.4 Possible contributors to persistence: structural, institutional, and interpretive factors

The persistence of this practice pattern, where it occurs, should not be interpreted as evidence of a single national culture or as the direct result of measured medico-legal pressure. Rather, the available literature supports a more cautious interpretation: several structural, institutional, and interpretive factors may make de-escalation difficult in some settings, even when evidentiary support for routine prolonged treatment is weak.

First, broad diagnostic labeling may enlarge the population exposed to inpatient management. Once the threshold for diagnosing threatened preterm labor is relatively permissive, more women may become candidates for admission, monitoring, and treatment, even when short-term delivery risk remains uncertain (2, 19).

Second, institutional variation points to the importance of local protocols and care environments. The regional and facility-level differences reported by Ogawa and colleagues imply that practice is not determined solely by patient condition, but may also be influenced by what individual hospitals regard as usual or prudent care (4).

Third, treatment options and clinical routines in Japan may have historically channeled practice toward ritodrine-centered management. Ogawa and colleagues noted that only ritodrine and magnesium sulfate were approved within the Japanese universal health insurance system for this clinical area, and also observed that until recently domestic guidance had not clearly specified a duration limit for *β*-agonist use (4). In such a setting, continuation of familiar therapy may be easier than withdrawal, particularly when clinicians face uncertainty about who will deteriorate and when.

Fourth, anxiety about sudden preterm birth may plausibly contribute to continuation, but this should be understood as an interpretive hypothesis rather than a directly measured causal pathway. Ogawa and colleagues suggested that “blind acceptance” of rule-of-thumb practice and anxiety among healthcare providers and pregnant women may contribute to the continued use of maintenance tocolysis (4). The workshop report by Murata and colleagues is also compatible with a wider regional pattern of cautious continuation, but it does not by itself establish the reasons for that pattern (5).

These factors do not make current practice evidence-based, nor do they prove why individual clinicians or institutions continue treatment. They may, however, help explain why de-implementation can be difficult when broad diagnostic labeling, local routines, limited therapeutic alternatives, and permissive guideline wording coexist. In this review, these elements are therefore treated as possible contributors to an evidence–practice gap, not as established causal determinants of clinician behavior.

## 6 Why Japanese guidelines need stronger negative recommendations

### 6.1 The problem is not only what guidelines recommend, but also what they fail to prohibit

The evidence–practice gap described in the preceding sections is sustained not only by clinical habit or institutional inertia, but also by the way standards are written. Guidelines do more than identify appropriate interventions. They also define the outer boundary of acceptable routine care. When that boundary is left indistinct, practices with weak evidentiary support may continue under the protection of interpretive ambiguity.

This point is particularly relevant in Japan because the JSOG/JAOG obstetrical guideline is explicitly intended to present standard obstetric diagnosis and management procedures that have reached professional consensus, and recommendations graded A or B are described as reflecting current standard care practices in Japan (21). In the section on preterm labor, the guideline contains several important management and safety elements. It recommends starting medication (e.g., tocolytic agents) when preterm labor is diagnosed, considering referral or transfer to a higher-level facility when necessary, administering magnesium sulfate for fetal neuroprotection, administering antenatal corticosteroids when preterm birth within 1 week is expected, and monitoring adverse events with consideration of dose reduction or discontinuation if symptoms improve (21). These statements indicate that the guideline recognizes both the need for acute obstetric management and the importance of safety monitoring. The underlying problem, therefore, is not complete absence of awareness.

This domestic context is also important in the Japanese-language 2023 JSOG/JAOG obstetrical practice guideline. In CQ302 on the diagnosis and management of threatened preterm labor, the guideline notes that ritodrine hydrochloride and magnesium sulfate are approved under the Japanese health insurance system and are widely used in Japan. It also states that evidence supports intravenous ritodrine within 48 h when the purpose is completion of one course of antenatal corticosteroids or maternal transfer to a facility capable of managing preterm infants, and that evidence does not show that long-term oral ritodrine maintenance therapy reduces delivery before 37 weeks or NICU admission (22). These statements show that recent domestic guidance has moved closer to a short-term, purpose-specific framing.

However, the wording remains more permissive than prohibitive. Although the guideline advises monitoring for adverse events and considering dose reduction or discontinuation when symptoms improve, it does not explicitly state that prolonged maintenance ritodrine should not routinely be used (21). That formulation may leave room for prolonged use to remain normalized as an acceptable discretionary option. In a clinical environment already shaped by broad diagnostic labeling, institutional routines, and uncertainty about preterm birth risk, such wording may be insufficient to support de-implementation. By contrast, some international guidance uses more direct negative language; for example, NICE explicitly states that betamimetics should not be offered for tocolysis (7).

Accordingly, the policy problem is not simply that Japanese guidance says the wrong thing. It is that current wording does not draw a sufficiently firm line around what should no longer be regarded as routine care. In the context of threatened preterm labor, failure to prohibit may function, in practice, as permission to continue.

### 6.2 Stronger wording could protect clinicians as well as patients

A stronger negative recommendation would not be punitive. On the contrary, it could provide protection for both patients and clinicians. For patients, the rationale is straightforward. As discussed in Section 4, prolonged ritodrine-based management has not shown convincing routine neonatal benefit, while maternal harms and neonatal complications have been documented (3, 9–18). Where benefit is uncertain and harm is credible, a guideline should do more than mention caution. It should help prevent avoidable exposure.

The clinician-protection argument should also be understood cautiously. This review does not directly measure clinicians’ legal concerns or emotional responses. Rather, it argues that when continuation has become a familiar response to uncertainty, and when domestic guidance does not clearly define the boundary of routine care, discontinuation may be perceived as a more institutionally exposed decision than continuation. This may be particularly relevant when threatened preterm labor is broadly labeled, when facilities vary in local norms, and when visible intervention is easily interpreted as active management (2, 4, 19, 20).

A clearer statement that prolonged maintenance ritodrine is not routine standard care could reduce this ambiguity. It may allow clinicians to withhold or discontinue long-term *β*-agonist treatment while remaining aligned with domestic professional guidance rather than appearing to depart from it. The practical question is often not whether long-term ritodrine is ideal, but whether its absence might later be interpreted as insufficient treatment. If the guideline explicitly states that non-use of prolonged maintenance tocolysis is consistent with standard care, the institutional burden of de-escalation may become lighter.

In that sense, stronger negative wording is not merely restrictive. It is an enabling tool for evidence-based restraint. It can protect patients from unnecessary treatment, while also protecting clinicians from the pressure to continue a practice chiefly because it remains ambiguously permissible.

### 6.3 Proposed principles for future JSOG wording

Future JSOG wording should therefore move from caution alone to clearer normative boundaries. Several principles follow from the evidence reviewed in this manuscript.

First, the guideline should distinguish explicitly between short-term goal-directed tocolysis and prolonged maintenance therapy. Short-term tocolysis, where it is employed, should be framed as a limited intervention for specific obstetric goals such as completion of antenatal corticosteroids, maternal transfer, or fetal neuroprotection, rather than as an open-ended strategy for routine pregnancy prolongation (6–8).

Second, the guideline should state directly that prolonged maintenance ritodrine should not routinely be used for threatened preterm labor. This statement should cover both continuation of intravenous therapy beyond the acute phase and routine step-down to oral maintenance treatment, unless an exceptional, clearly documented reason is present (9–12).

Third, the guideline should clarify that the absence of long-term tocolysis does not constitute deviation from standard care when maternal and fetal status are appropriately assessed and monitored. This point is crucial if the aim is not only to discourage overuse, but also to protect clinicians who practice according to evidence rather than custom.

Fourth, exceptional continuation beyond the acute phase, if judged necessary, should be treated as the exception rather than the norm. In such cases, the indication, intended objective, anticipated duration, and risk–benefit reasoning should be documented explicitly. This would preserve clinical discretion without allowing routine maintenance therapy to persist under vague justification.

Finally, future wording should align the management of threatened preterm labor more clearly with the conceptual distinction outlined earlier in this review. Broad symptomatic presentations without clear evidence of imminent delivery should not automatically trigger a prolonged intervention package. Stronger diagnostic precision and stronger negative recommendations on maintenance ritodrine are therefore complementary, not separate, reforms.

For Japan, the central guideline question is no longer whether prolonged ritodrine warrants caution. It is whether guidance should continue to leave room for a practice that the evidence does not support as routine care. On the basis of the literature reviewed here, a clearer negative recommendation is justified.

## 7 Actionable recommendations

### 7.1 For guideline developers

The first priority for guideline developers is to convert general caution into operationally clear recommendations. Future JSOG wording should state explicitly that prolonged maintenance ritodrine should not routinely be used for threatened preterm labor, and should distinguish this clearly from short-term tocolysis used for defined obstetric goals such as completion of antenatal corticosteroids or maternal transfer (6, 7). The intended gestational range, clinical purpose, and expected duration of treatment should be specified in concrete terms rather than left to broad interpretation (21).

Guideline language could operationalize this distinction as a stepwise pathway. First, women presenting with symptoms suggestive of preterm labor should be assessed for gestational age, contraction pattern, cervical change, membrane status, maternal or fetal indications for admission, and, where feasible, objective short-term risk markers such as transvaginal cervical length or fetal fibronectin. Second, when short-term delivery risk appears low and no maternal or fetal indication for admission is present, routine hospitalization, bed rest, prolonged intravenous ritodrine, and oral maintenance therapy should not be initiated as default care; management may instead include counseling, safety-netting, and planned reassessment or outpatient follow-up. Third, when short-term delivery risk is high, or when antenatal corticosteroids, maternal transfer, or magnesium sulfate for fetal neuroprotection is indicated, tocolysis should be framed as a short-term, goal-directed intervention rather than an open-ended treatment. Fourth, reassessment should be required within 24–48 h or once the acute objective has been achieved. Treatment reduction or discontinuation should be the default when contractions settle, objective risk decreases, transfer or corticosteroid administration has been completed, or adverse effects emerge. Finally, continuation beyond the acute window should be exceptional and should require documentation of the indication, intended objective, expected duration, and risk–benefit reasoning (4, 6, 7, 21).

### 7.2 For hospitals and obstetric departments

Hospitals and obstetric departments should translate revised principles into local protocols. These protocols should define the criteria for starting tocolysis, the short-term objective of treatment, a predefined reassessment point at 24–48 h or after completion of the acute objective, criteria for treatment reduction or discontinuation, criteria for continued admission or transfer, and documentation requirements for exceptional continuation (3, 4, 21). A protocol of this kind would reduce unnecessary variation between institutions and limit the discretionary drift by which short-term treatment becomes prolonged routine care (4). Such protocols should also make clear that de-implementation of routine prolonged hospitalization or maintenance ritodrine is not equivalent to minimizing care for high-risk patients; rather, it requires distinguishing women who need continued inpatient management from those for whom reassessment, treatment reduction, discharge planning, or outpatient follow-up is appropriate. This approach would make de-escalation a normal pathway within care rather than an exceptional decision by an individual clinician. Departments should also develop shared explanatory language for clinicians, midwives, and neonatal teams so that maternal counseling is consistent across providers. Where possible, obstetric and NICU teams should agree in advance on how to explain that non-use of long-term ritodrine may reflect evidence-based management rather than therapeutic inaction. Such alignment could help reduce “point-of-care defensiveness”, in which treatment is continued partly because stopping it feels institutionally or emotionally unsafe (4, 19, 21).

### 7.3 For future research and surveillance

Future research should focus less on re-testing prolonged maintenance tocolysis as a general concept and more on monitoring whether practice changes after guideline clarification. National inpatient and claims databases have already shown that Japanese practice can be measured at scale (3, 4, 19). These platforms should be used prospectively to assess whether stronger domestic recommendations are followed by reductions in prolonged ritodrine use, shorter admissions, or narrowing of regional and facility-level variation (4, 19).

Safety surveillance should also continue. Maternal monitoring should include severe cardiopulmonary complications and mortality signals associated with ritodrine exposure (14, 15). Neonatal surveillance should include hypoglycemia and electrolyte complications, particularly where ritodrine and magnesium sulfate are used in close temporal proximity (16–18). If de-implementation is pursued, these outcomes should be tracked before and after change so that clinicians and policymakers can evaluate both safety and acceptability.

Finally, diagnostic standardization requires dedicated study. The broad and variable use of the threatened preterm labor label in Japan has been a major driver of the overall evidence–practice gap (2, 19). Future work should therefore examine how symptom-based presentations can be stratified more consistently, which objective markers are most feasible in Japanese practice, and how revised diagnostic definitions influence treatment decisions. Without better diagnostic precision, even stronger recommendations on ritodrine may be only partially effective.

## 8 Discussion

This review has argued that the management of threatened preterm labor in Japan represents a persistent evidence–practice gap rather than a narrow disagreement over one drug. The problem begins at the diagnostic level. In Japan, the concept of threatened preterm labor appears to be applied across a relatively broad clinical spectrum, allowing many women with symptomatic presentations but uncertain short-term delivery risk to enter a more intensive treatment pathway (2, 19). This contrasts with contemporary international approaches, in which diagnosis and intervention are more explicitly linked to objective risk assessment and to the likelihood that short-term treatment will alter clinically meaningful outcomes (6–8).

Within that pathway, prolonged ritodrine-based management has remained common despite limited evidentiary support for routine maintenance therapy (3, 4). The available literature does not show sufficient benefit to justify long-term tocolysis as standard care, while maternal harms and neonatal complications have been documented with increasing clarity (3, 9–18). For this reason, the central concern is not merely whether ritodrine can still be found in practice, but whether a long-term *β*-agonist-centered approach continues to occupy a routine place in care without the evidentiary balance normally required for such a role.

At the same time, the persistence of practice should not be interpreted as resulting from a single cultural or medico-legal cause. As shown in the preceding sections, continued use of prolonged ritodrine-based management may be related to the interaction of broad diagnostic labeling, institutional routines, regional and facility-level variation, limited therapeutic pathways, and guideline wording that does not clearly define the boundary of routine care (2–5, 19–22). The cultural and medico-legal explanations discussed in this review should therefore be understood as policy interpretations and possible contributors, not as directly measured determinants of clinician or patient behavior.

The implications therefore extend beyond Japan alone. This case may illustrate a broader policy problem that can arise in many health systems: evidence may not automatically displace embedded practice when diagnostic categories are expansive, treatment routines are institutionalized, and guidance leaves room for continuation by default. In that sense, threatened preterm labor in Japan is not only a local controversy, but also a useful example of how evidence–practice gaps may be maintained in obstetric care.

Several limitations should be acknowledged. This article is a narrative policy and practice review rather than a systematic review, and the sources were selected to support a focused analysis rather than an exhaustive inventory of all available literature. No formal risk-of-bias assessment, GRADE evaluation, or quantitative evidence grading was performed. The review relied primarily on English-language publications and major international guidance documents, although the Japanese-language 2023 JSOG/JAOG obstetrical practice guideline was also reviewed to clarify current domestic wording (22). Other Japanese-language literature, local institutional protocols, and unpublished practice patterns may not have been fully captured. In addition, Japanese practice is heterogeneous, and the patterns described here should not be interpreted as applying uniformly to all regions, facilities, or clinicians. Duration thresholds also differed across studies, including treatment beyond 48 h, treatment for four days or more, and very prolonged inpatient intravenous therapy lasting 28 days or more; these thresholds should not be interpreted as equivalent exposure definitions. Much of the evidence on real-world practice comes from observational database studies, claims analyses, and case-based literature, which can identify associations and practice patterns but cannot fully establish causality or separate the independent effects of diagnosis, hospitalization, bed rest, and ritodrine exposure. The medico-legal and cultural explanations proposed in this review should therefore be understood as policy interpretation rather than direct empirical measurement of clinician or patient attitudes.

For that reason, clearer guideline wording is not a secondary editorial matter. It is a practical mechanism for changing the boundary of routine care, protecting patients from unnecessary exposure, and enabling clinicians to de-escalate treatment without appearing to depart from standard practice (21). If threatened preterm labor in Japan is to be managed more consistently with current evidence, reform must address diagnosis, institutional practice, and guideline language together. The Japanese experience therefore suggests a wider lesson: in obstetrics, closing an evidence–practice gap requires not only better evidence, but also clearer norms about when not to intervene.
